# The external morphology of *Austroplatypus incompertus* (Schedl) (Coleoptera, Curculionidae, Platypodinae)

**DOI:** 10.3897/zookeys.56.521

**Published:** 2010-09-17

**Authors:** Deborah S. Kent

**Affiliations:** Forest Resources Research, Science & Research, Industry & Investment New South Wales, P.O. Box 100 Beecroft NSW 2119 Australia

**Keywords:** Curculionidae, Platypodinae, Austroplatypus, Australia, external morphology, larvae, sexual dimorphism, latitudinal cline

## Abstract

Previous descriptions of adult Austroplatypus incompertus (Schedl) are completed by the addition of descriptions and illustrations of the adults and, in particular, their maxillary palps. I describe and illustrate the non-adult phases of the life cycle and provide a key to the larval instars. The sexual dimorphism of Austroplatypus incompertus is atypical and includes a latitudinal cline which obeys Bergmann’s rule. The taxonomic position of the genus within the Platypodinae is clarified. Platypus incostatus Schedl is recognised as the male of the species, and hence a new synonym of Austroplatypus incompertus.

## Introduction

Studying and accurately describing the external morphology of insects is important because it is the external details that characterise and give indications of the animals’ biology, ecology and social behaviour. In addition, insects are often identified solely by external adult morphology and this is reflected in the formal taxonomic descriptions of most species.

Studies of the external morphology of platypodines have generally concentrated on taxonomic research and consequently have mainly dealt with descriptions of specific parts of the external morphology of adults. In contrast, relatively few papers (Hogan 1948, Browne 1961, Roberts 1962, Santoro 1957, 1965, Candy 1990) have described all the developmental stages of a particular species.

The lack of comprehensive morphological descriptions within the platypodines is typified by Austroplatypus incompertus (Schedl). This species was discovered in the early 1950s and named in 1968. Describing and identifying it has been problematic as specimens are unusually difficult to collect. Adult beetles are not attracted to light and the only way to obtain all developmental stages is to cut them from living trees. Consequently there has been a dearth of material for description or comparison. In fact, until the current study, no well-documented collection of both adult and larval Austroplatypus incompertus material had been made. This has no doubt contributed to the small number of papers dealing with the taxonomy and biology of Austroplatypus incompertus (Schedl 1968, Campbell 1969, Browne 1971, Harris et al. 1973, 1976). There are even fewer studies of the immature stages. No descriptions exist of the eggs or pupae, and although it is generally accepted that there are five larval instars (Harris et al. 1973, Wright and Harris 1974; Harris et al. 1976), only the final instar has been described (Browne 1972). There are no records of any morphological differences between instars or any measurements of head capsule widths, an important method for differentiating instars.

The research reported in this paper had two broad aims. The first was to verify and complement existing descriptions of the external morphology of Austroplatypus incompertus. This necessitated descriptions of the egg, larval instars and the pupa as well as a description of adult sexual dimorphism and the adult maxillary palps. In addition immature stages and adults were illustrated with line drawings and scanning electron micrographs.

An integral part of these descriptions were measurements such as head capsule widths and adult body lengths. However, such measurements are problematic if variation exists among populations. The increase in mature larval head capsule width of Platypus subgranosus Schedl from north to south (Candy 1990) illustrates the need to take measurements over a wide geographical range where possible. Thus, size variation was also examined for a number of factors, viz. geographical location for larvae and adults and sex and host tree species for adults.

The second aim was to integrate the new material presented here with existing taxonomic descriptions and discuss the current taxonomic position of Austroplatypus incompertus.

## Material and methods

The source materials for this paper were voucher specimens collected during the study as well as material already held in the Forestry Commission of NSW Insect Collection (FCNI). In their entirety these specimens encompassed the currently known geographical and host tree species range of Austroplatypus incompertus (Kent 2008a). Type material of both Austroplatypus incompertus (National Museum of Victoria) and Platypus incostatus (British Museum) was also examined.

### Collections examined

AMAustralian Museum

ANICAustralian National Insect Collection

BMThe Natural History Museum, London

FCNIForestry Commission of NSW Insect Collection

NMVNational Museum of Victoria

SAMSouth Australian Museum.

## Material examined

Unless otherwise noted all specimens mentioned below are part of FCNI.

### Austroplatypus incompertus

(Schedl)

#### Holotype female:

New South Wales: Eden, 23.x.1953 (NMV), LH Bryant. Ex. Eucalyptus sieberana F. Muell. (= Eucalyptus sieberi L. Johnson).

#### Paratypes:

Victoria: Woodhouse Creek, N of Omeo, xi.1964 (1♀ NMV); near Omeo, xi.1965 (2♀ NMV). (Note: vthe distribution of the paratypes examined at NMV does not agree with Schedl (1968) which lists 2 paratypes from Woodhouse Creek and 1 near Omeo).

#### Other material examined:

New South Wales: Dorrigo, 23.iii.1954 (1♀) [Note: same information as Holotype of Platypus incostatus]; Styx River State Forest, 24.ix.1992 (11♀), 16.x.1992 (7♀); 20.i.1993 (1♀,1♂); Mt Boss State Forest, 27.iii.1958 (2♀), Bellangry State Forest, 12.xi.1965 (5♀); 7.xii.1988 (13♀); Bellangry Timber Mill, Wauchope, 9.xii.1988 (1♀); Manning River National Forest, Taree, 10.viii.1965 (5♀), 8.ix.1965 (2♀), 9.ix.1965 (1♀), 19.vi.1967 (3♂); Coopernook State Forest, 11.viii.1965 (1♀); Ourimbah State Forest, Wyong, 20.xi.1984 (1♀),18.iii.1988 (6♀,5♂), 13.x.1988 (1♀), 22.xi.1988 (22♀), 17.i.1989 (1♀), 24.ii.1989 (3♀,1♂), 16.vi.1989 (11♀,1♂); Mt Wilson, Blue Mtns, iv.1986 (3♂ AM); Banshea State Forest, near Oberon, 7.x.1965 (5♀), 18.iii.1970 (3♀,1♂); Cumberland National Forest, West Pennant Hills, 19.viii.1965 (1♀), 30.viii.1965 (1♀), 30.ix.1965 (1♀), 28.x.1965 (3♀), 12.iv.1967 (1♀), 19.iv.1967 (1♀,1♂), 26.ix.1967 (1♀), 6.xii.1967 (1♀); iii-v.1968 (1♀,6♂), 8.iv.1969 (1♀,4♂), 30.iii.1988 (1♂), 5.iv.1988 (1♀), 8.iv.1988 (2♀), 14.iv.1988 (15♀,1♂), 19.iv.1988 (1♀), 20.iv.1988 (1♀), 21.iv.1988 (1♂), 13.xii.1988 (2♀), 23.ii.1989 (1♀), 1.iii.1989 (1♀), 30.iii.1989 (23♂), 3.iv.1989 (11♀,2♂), 4.iv.1989 (1♀,4♂), 5.iv.1989 (1♀,1♂), 10–12.iv.1989 (9♀,8♂), 11.iv.1989 (1♀), 2.iv-6.v.1990 (75♀,55♂), 14.vi.1990 (4♀), 25.x.1990 (3♀), 18–19.iv.1991 (1♂), 20–21.iv.1991 (1♀,1♂); 4–14.iv.1991 (3♂), 4–24.iv.1991 (24♀,13♂), 30.iv-1.v.1991 (1♀), 4–5.iv.1992 (1♂), 6.iv.1992 (1♂), 7.iv.1992 (2♀,6♂), 6–7.iv.1992 (3♀,11♂), 7–8.iv.1992 (2♂), 11–12.iv.1992 (4♀,11♂), 13.iv.1992 (17♀, 38♂), 14.iv.1992 (19♀,1♂), 15.iv.1992 (6♀,7♂), 15–18.iv.1992 (9♀), 18.iv.1992 (3♀,2♂), 18–21.iv.1992 (2♀,1♂), 24.iv.1992 (1♂), 25–28.iv.1992 (9♀,4♂), 29.iv.1992 (1♀), 30.iv.1992 (4♀,1♂), 1–11.v.1992 (12♀,1♂), 6–8.v.1992 (1♀); Broughton’s Lookout, 15 kms S of Wombeyan Caves, 27.viii.1979, (1♂ AM); Nullica State Forest, Eden, 19.xi.1991 (1♀); Nalbaugh State Forest, Bombala, 21.xi.1991 (7♀); Bondi State Forest, Bombala, 21.x.1965 (4♀); Bombala, 13.iii.1991 (3♀,6♂); Eden, 23.x.1953 (1♀)[Note: same information as Holotype], 25.vii.1989 (4♀); Naghi State Forest, Eden, 20.x.1965 (4♀).

Victoria: Lightning Creek, N of Omeo, viii.1965 (1♂ NMV); Woodhouse Creek, near Omeo, viii.1965 (2♀, NMV); Swifts Creek, 20 miles (32 km) S of Omeo, 1966 (2♀ NMV; 2♀ SAM; 1♀ ANIC), 14.iv.1967 (3♀,1♂).

### Platypus incostatus

Schedl

#### Holotype male:

New South Wales: Dorrigo, 23.iii.1954 (BM), J Cartwright. Ex. Eucalyptus laevopinea R.T. Baker.

### Specimen preparation – General

All specimens of immature life stages were fixed to preserve their shape and size and thus ensure that descriptions and measurements were accurate and comparable. Eggs, larvae and pupae were fixed in KAA [kerosene (7%), glacial acetic acid (16%) and 95% ethanol (77%)] for 5–30 minutes depending on size and developmental stage; eggs and small larvae required the shortest fixing period and the fifth instar and pupae the longest. The fixed specimens were then passed through immersion stages of several hours each in 90% and 85% ethanol before eventual permanent storage in 80% ethanol. Adults were killed either by freezing or by immersion in 80% ethanol and then air-dried and mounted on card points.

### Specimen preparation - Scanning electron microscopy

For scanning electron microscopy, specimens were fixed in 2% glutaraldehyde in phosphate buffer (pH 6.9) for 24 hours, transferred through a series of increasing ethanol concentrations and then stored in absolute ethanol until coating. Just before coating, the specimens were placed in either ethyl acetate or acetone and then critical-point dried, after which they were sputter-coated with platinum or gold.

### Observations

Observations of all life cycle stages of the beetle were made using stereo-dissecting and compound microscopes and a Cambridge S120 scanning electron microscope with a Robinson detector. These observations formed the basis for the descriptions of the external structures. The morphological terminology for larval descriptions follows that used by Browne (1961, 1972) and Roberts (1962).

### Sex determination of adults

Adult beetles were sexed based on external morphological differences (Kent 2001): females by the presence of mycangia and a spined elytral declivity; males by the absence of mycangia and presence of simple rounded elytral apices.

### Measurements

Both adult and larval measurements were made using a stereo-dissecting microscope fitted with a scaled graticule. One measurement was recorded for larvae: the width of the head capsule at its widest point. Three measurements were recorded for adults: dorsal prothorax length (measured along middle of the prothorax) and width (measured at its widest point, the posterior edge of the femoral emargination), and elytral length. Although total length is commonly reported in taxonomic descriptions, this measurement proved to be unreliable due to post-mortem head deflection and was therefore not recorded. Some adult beetles could not be measured because of their position on the card mounts.

### Statistical analysis

**Figure 1.**
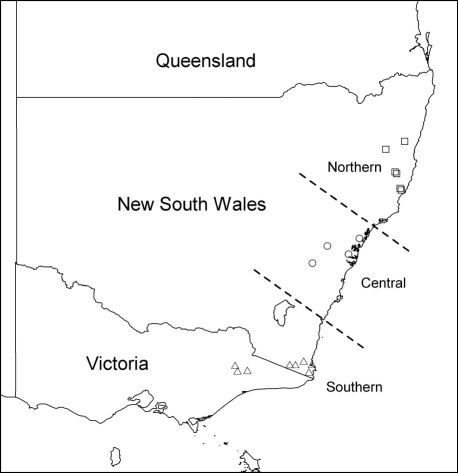
Latitudinal distribution of adults and larvae from which measurements were taken.

Analysis of variance (ANOVA) was carried out on the measurements of the fifth instar larval head widths to determine whether there was a difference in specimens from different localities (the localities from which study material was derived fell naturally into three well separated latitudinal groups - Fig. 1). Similarly, measurements of adult beetles were analysed using ANOVA to determine whether there were differences between sexes, or between adult females from different localities or different host species (there being too few males from the range of localities and hosts to permit analysis). The analyses of females were restricted to prothorax length, as this was the most repeatable measurement. Where appropriate, Post hoc Tukey Honest Significant Difference (HSD) multiple comparison tests were performed to determine which means differed significantly.

## Results

### Descriptions

#### Egg

(n = 40) length 0.70 mm ± 0.06, width 0.45 mm ± 0.04 (SD). Elongate, translucent white, shiny, without obvious sculpturing.

#### First instar larva

**Figure 2.**
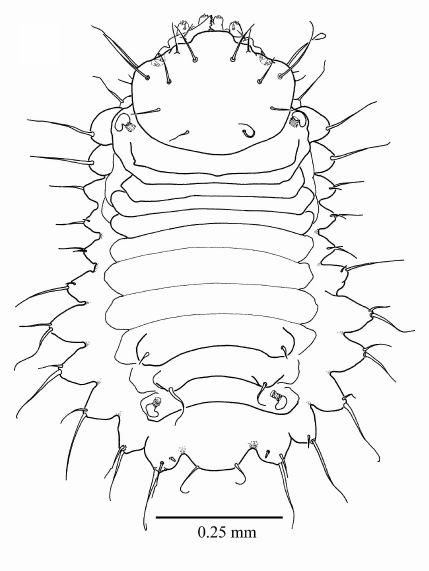
First instar larva, habitus, dorsal.

**Figure 3.**
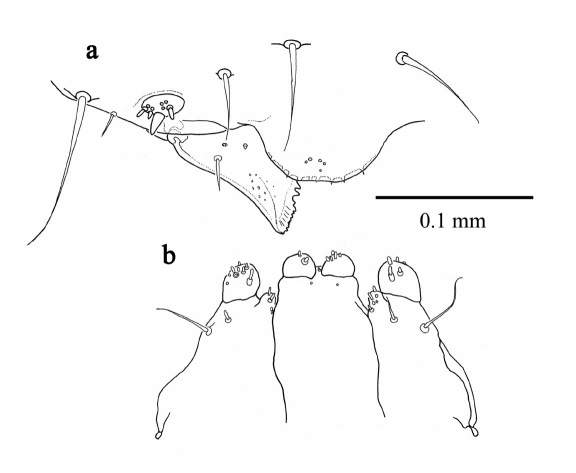
First instar larva a antenna, labrum and mandible, dorsal b maxillae and labium, dorsal.

(Figs 2, 3a, b) length 1.183 mm ± 0.2 (SD) (n = 5), maximum body diameter much smaller than that of gallery. Maximum width on 5th abdominal segment. Body hyaline, white, shape ovoid and hump-backed dorsally. Head exserted, width of head capsule 0.303 mm ± 0.012 (n = 10), wider than long, greatest width over bulbous antero-lateral margins. Head setae prominent, 1 posteriorly to each antero-lateral margin, nearly twice as long as any other. Antennae small, one each side of epicranium in unpigmented portion between mandibular condyles (Fig. 3a). Mandibles lightly sclerotised and with comb-like teeth (Fig. 3a). Maxillary palpi 1-segmented; labial palpi 1-segmented (Fig. 3b). Meso- and metathoracic segments each enlarged into pseudopods. All thoracic segments bearing a single prominent lateral seta on each side. Abdominal tergites 6 and 7 each bear a single prominent seta on a dorsolateral protuberance on each side. All sternites with lateral protuberances, each bearing two setae, those of segments 5–9 enlarged into pseudopods (Fig. 2). Only two pairs of spiracles, one on prothorax and the second on abdominal segment eight.

#### Second instar larva

**Figure 4.**
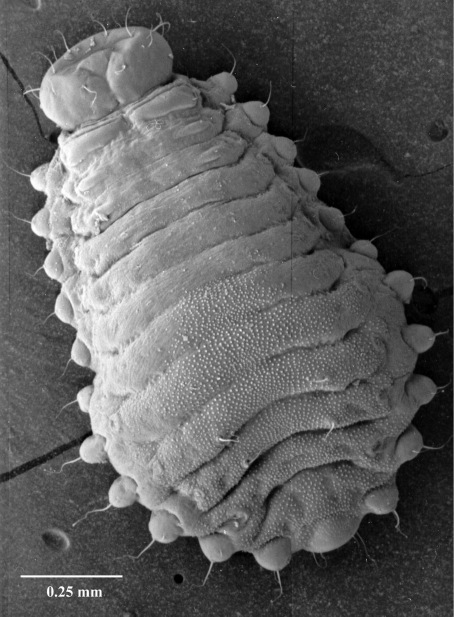
Second instar larva, habitus, dorsal.

(Fig. 4) slightly larger than first instar, but same general form. Head more elliptical, less angular than first instar. Head capsule width 0.353 mm ± 0.028 (SD) (n = 11). Mandibles similar. The main difference between the first two instars is the presence of nine pairs of spiracles, one each on the prothorax and one each on eight abdominal segments.

#### Third instar larva

**Figure 5.**
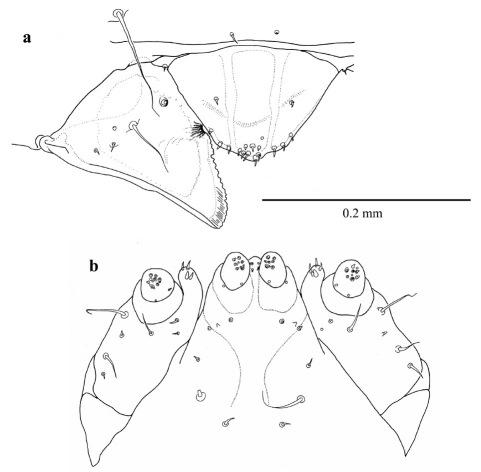
Third instar larva a mandible, labrum and epistoma, dorsal b maxillae and labium, ventral.

(Fig. 5a, b) slightly larger than the second instar, but still smaller than gallery diameter. Head capsule width 0.581 mm ± 0.077 (SD) (n = 32). Body still hump-backed but more flattened ventrally and not as translucent as first two instars. Head distinctly narrower than prothorax and more rounded in shape in comparison to the first two instars. Pseudopods no longer as prominent. Spiracles as in second instar. Mouthparts as in Fig. 5a, b.

#### Fourth instar larva

**Figure 6.**
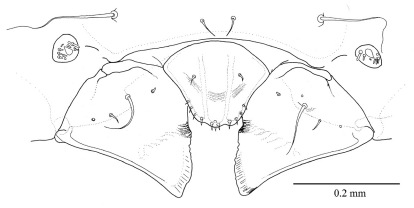
Fourth instar larva, antennae, mandibles, labrum and epistoma, dorsal.

(Fig. 6) body stout, more or less closely fitting the galleries. Head clearly narrower than width of pronotum. Pseudopods not evident. Head capsule slightly wider than long, width 0.868 mm ± 0.071 (SD) (n = 49). Labrum and mandibles as in Fig. 6. Mandibles similar to third instar, only slightly chitinized and still bearing small teeth along apical and subapical edges. Pronotum lacking any chitinized pattern of ridges (see below).

#### Fifth instar larva

**Figure 7.**
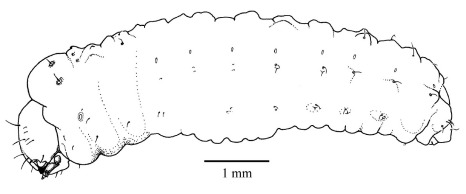
Fifth instar larva, habitus, lateral.

**Figure 8.**
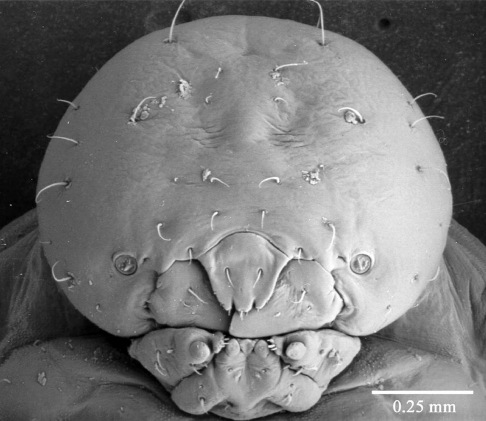
Fifth instar larva, head, ventral.

**Figure 9.**
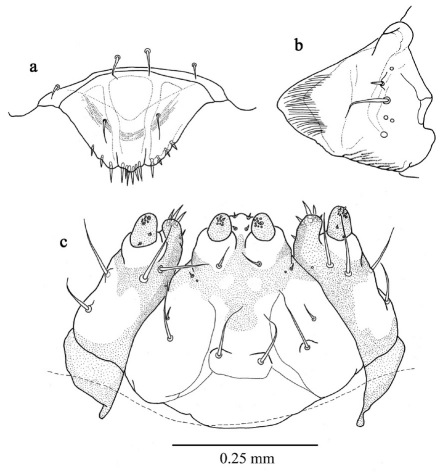
Fifth instar larva **a** labrum and epistoma, dorsal **b** mandible, dorsal **c** maxillae and labium, ventral.

**Figures 10, 11.**
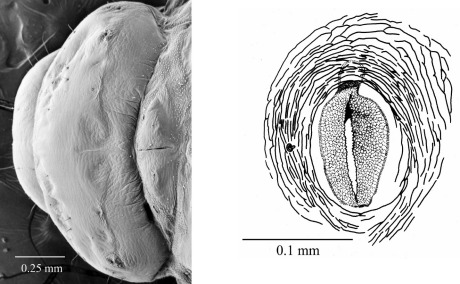
**10** Fifth instar larva, pronotum, dorsal **11** Fifth instar larva, thoracic spiracle.

(Figs 7, 8, 9a, b, c, 10, 11) see Browne (1972) for detailed description. The following is supplementary to Browne’s description. Head capsule width 1.121 mm ± 0.086 (SD) (n = 547). Figures 8 and 9a, b, c illustrate head with details of labrum, mandible and labium, respectively. Mandibles heavily chitinized, bluntly pointed and lacking teeth. Pronotum pattern with two pairs of setae, the lateral seta of each pair socketed and surrounded by irregular chitinized ridges, the medial seta surrounded by lighter chitinized irregular ridges (Fig. 10). Spiracles (Fig. 11) ovate with single, short, dorsally directed air tube; peritreme surrounded by cuticular wrinkles. Thoracic spiracles larger than abdominal ones.

#### Five distinct larval instars are morphologically discernible using the following key

Five distinct larval instars are morphologically discernible using the following key

**Table:** 

1.	Body ovoid, trapezoidal, rhomboidal, hump-backed dorsally; body width distinctly less than diameter of gallery (Figs 2, 4)	2
–	Body elongate, not markedly hump-backed; body width almost the same as that of the gallery (Fig. 7)	4
2.	Prominent pseudopods on meso- and meta-thoracic segments and on abdominal segments 5–8, head broader than long (Figs 2, 4)	3
–	Pseudopods not very prominent on any body segment, head more round	third instar
3.	Only two pairs of spiracles, both the same size, one pair on prothorax and one on abdominal segment 8, head very broad, transversely oblong (Fig. 2)	first instar
–	Nine pairs of spiracles, one pair on prothorax and one pair on each side of first eight abdominal segments, head more elliptical (Fig. 4)	second instar
4.	Pronotum lacking any brown chitinized pattern; mandibles slightly chitinized with teeth on cutting margin (Fig. 6)	fourth instar
–	Pronotum with brownish chitinized patterned consisting of two pairs setae, surrounded by irregular ridges (Fig. 10); mandibles heavily chitinized, apex bluntly pointed and free of small teeth (Fig. 9b)	fifth instar

#### Pupa

**Figure 12.**
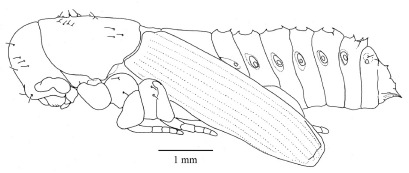
Pupa, habitus, lateral.

**Figure 13.**
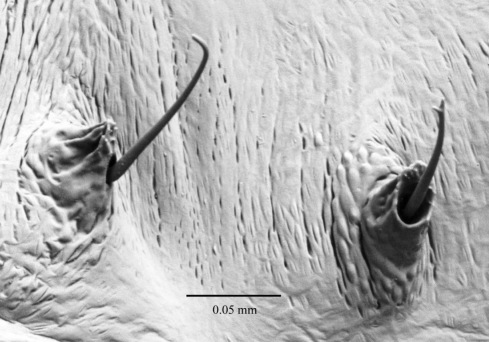
Pupa, thoracic setae, dorsal.

(Fig. 12) cuticle white and glabrous, setae coarse, arising laterally from an armed tubercle (Fig. 13), larger and more numerous on head and prothorax than on abdomen. Rostrum not reaching fore coxae. Antennal club smooth. Sex indeterminable until the darkening of the cuticle of the young adult appears through the pupal skin. At this point females can be identified by the appearance of mycangia in the centre of the prothorax.

#### Adults

**Figure 14.**
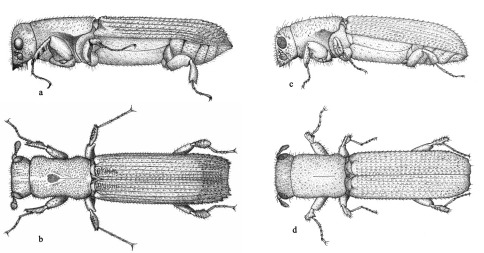
Adult female (**a&b**) and male (**c&d**) (lateral and dorsal respectively).

(Fig. 14a, b, c, d) have the typical elongate cylindrical form of platypodines, with a length of approximately 6 mm and a diameter of approximately 2 mm. All types examined at NMV were females. This is in contradiction to Schedl’s tentative assignment of them as all male (Schedl 1968).

Schedl (1972b) described Platypus incostatus as a new species closely allied to Austroplatypus incompertus, from a single specimen of unspecified sex from Dorrigo, N.S.W., collected on 23 March 1954. Following the suggestion of R.A. Beaver (pers. comm.) that the holotype of Platypus incostatus described by Schedl might in fact be a male Austroplatypus incompertus I examined the holotype (held by the Natural History Museum, London) and confirmed that it is a male Austroplatypus incompertus. Interestingly, the FCNI collection contains a single female Austroplatypus incompertus collected from the same locality on the same day, but Schedl evidently never saw this specimen.As the name Platypus incompertus predates that of Platypus incostatus, the latter becomes a synonym.

Provided one is aware of these problems Schedl’s 1968 and 1972b descriptions of adult beetles are fairly complete. Since they have already been published do not require repeating in this paper. Inadequacies in the original description of the adult head (Schedl 1968) were remedied by Browne (1971). In addition the mycangia in female Austroplatypus incompertus are described in detail and illustrated in Kent (2008b).

#### Maxillary palps

**Figure 15.**
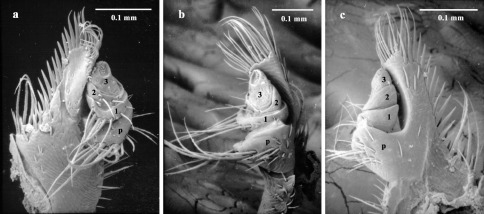
Adult maxillary palp **a** ventral **b** lateral **c** dorsal (segments numbered 1–3 and palpiger – p).

(Fig. 15a, b, c) of adult Austroplatypus incompertus are three segmented. This is in contrast to a previous report that they have four segments (Browne 1971). Browne’s error appears to have resulted from mistaking part of the palpiger as an additional segment (Zimmerman 1994, Kuschel 1995, pers. obs.). Because of the importance this misidentification has played in the taxonomic debate regarding whether platypodines and scolytines should be placed within or outside of Curculionidae (Wood 1973), the palp is illustrated here in detail for the first time. This figure shows the palp in a sequence of views, the ventral one showing a superficial resemblance to a four segmented condition. This resemblance disappears when the palp is rotated to the dorsal view.

### Sexual dimorphism

**Figure 16.**
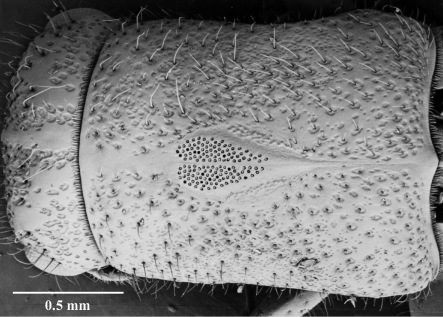
Mycangia of female.

**Figure 17.**
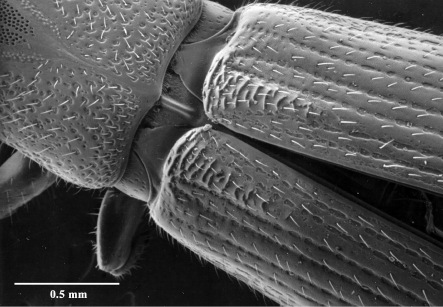
Elytral ridges of female.

The sexes are dimorphic in Austroplatypus incompertus with the most obvious difference being the shape and sculpturing of the elytra. In the female the elytral declivity is abrupt and armed with prominent spines, while in the male the elytral apices are more rounded with only very small spines. This difference between the sexes is easily discernible with the naked eye and can be used to sex individuals in the field. Additional differences between the sexes can be seen using a stereo-dissecting microscope:

–	The presence in the female of mycangia in the centre of the prothorax (Fig. 16) and their absence in the male;

–	The presence in the female of a series of ridges at the base of the elytra (Fig. 17) and their absence in the male. The ridges are located between the 3rd and 4th interstices and form a series of backwardly directed ridges, twice as wide at the anterior edge (straddling both the 4th and 3rd interstices) and tapering to half that (just the 3rd) for approximately a sixth of the total length of the elytra.

### Size differences

#### Larvae

Only fifth instar larvae were present in sufficient numbers to allow analysis of variance. There were highly significant differences in head capsule width among the different latitudinal groups [P < 0.001, DF = (2, 543)] with the width increasing from north to south (Table 1).

**Table 1.** Head capsule width of fifth instar for different latitudinal groups.

Latitudinal group	n	Mean head capsule width (mm)
Northern	84	1.015 ^a^
Central	292	1.087 ^b^
Southern	171	1.230 ^c^

#### Adults

##### Between sexes

Although both the prothorax and the elytra were measured, the prothorax proved to be better suited to regular measurement because of its flatness, especially along its length. Measurements based on the prothorax were also less likely to be subject to error, compared to elytral length, as the latter can be difficult to measure if the elytra are opened after the death of the specimen. Even so, the results of the analysis of the three different measurements pooled across all hosts and localities showed that all were highly significantly different between the sexes [prothorax length P < 0.001, DF = (1, 604); prothorax width P < 0.001, DF = (1, 603) and elytral length P < 0.001, DF = (1, 604)], with males being smaller than females.

Because all three measurements were significantly different between the sexes but prothorax length was the most suitable and reliable measurement, subsequent analyses were restricted to this variable. Males were present in too few numbers across all localities and hosts to permit analysis.

##### Between localities

There was a highly significant size difference between female beetles from the different latitudinal groups [P < 0.001, DF = (2, 428)] with prothorax length increasing from north to south (Table 2).

**Table 2.** Mean prothorax length of Austroplatypus incompertus females for each of the three latitudinal groups.

Latitudinal group	n	Mean head capsule width (mm)
Northern	70	1.621 ^a^
Central	314	1.745 ^b^
Southern	47	1.872 ^c^

##### Between hosts

There was also a highly significant difference between beetles from the different host tree species [P < 0.001, DF = (8, 418)], and Post hoc Tukey HSD multiple comparisons placed the hosts into three groups (Table 3).

**Table 3.** Mean prothorax length of Austroplatypus incompertus females for each of the host tree Eucalyptus species.

Host species	n	Prothorax length (mm)	Host distribution
Eucalyptus andrewsii	16	1.533 a	Northern tablelands of NSW & adjacent areas of Queensland
Eucalyptus cameroni	2	1.630 a b	Northern tablelands & ranges of NSW
Eucalyptus laevopinea	15	1.681 b	Central & northern tablelands of NSW and immediately adjacent areas of Queensland
Eucalyptus pilularis	300	1.723 b	Coastal NSW and southeast Queensland
Eucalyptus obliqua	33	1.811 c	Northern tablelands & south coast of NSW, coast & ranges Victoria, Mt Lofty Ranges South Australia and Tasmania.
Eucalyptus agglomerata	36	1.817 c	Central & southern coast of NSW and adjacent areas of Victoria
Eucalyptus fastigata	6	1.856 c	Tablelands, ranges & coastal escarpments of NSW and adjacent parts of Victoria
Eucalyptus sieberi	16	1.879 c	Tablelands & coast of NSW and eastern Victoria
Eucalyptus delegatensis	3	1.914 c	Southern ranges of NSW and eastern Victoria

## Discussion

### Immature stages

Five distinct larval instars could be distinguished on the basis of their morphology and their head capsule widths, as is the case with other platypodines (Browne 1961, 1972, Hogan 1948, Roberts 1960, 1962, 1968, Candy 1990). The external morphology of the fifth instar larval stage of Austroplatypus incompertus is characterised by the design of the prothorax pattern which separates it from the larva of Dendroplatypus, which it most closely resembles (Browne 1972). Browne (1972) also split Austroplatypus from the majority of platypodines based on his observation that the anterior notches of the labrum are shallow. This study found that the notches in fifth instar larvae were not shallow as illustrated by Browne (1972) but more deeply and narrowly notched (Fig. 8). This level of detail is only seen in scanning electron micrographs. Preserved specimens and slide preparations show the labrum with shallow notches because these preparation methods appear to result in the closure of the gaps between the notches (Fig. 9a).

### Sexual dimorphism

Sexual dimorphism in Austroplatypus incompertus is reversed, compared with the situation in other platypodines. Males are significantly smaller than females, females have elytral modifications, in the form of an elytral declivity modified for both cleaning and defence, which males do not, and only females possess mycangia. In most platypodines, the sexes are similar in size or the male is only slightly smaller, males alone possess elytral modifications or modifications are much more strongly developed in males, and males either have no mycangia or a reduction in the number of mycangia compared with females (Chapuis 1865, Strohmeyer 1914, Hogan 1948, Milligan 1979, Roberts 1960, Wood 1993, Beaver and Liu 2007). Wood (1993) linked these external modifications, or lack of them, to the typical Platypodinae monogynous mating system and male initiated gallery system. This is consistent with the situation in Austroplatypus incompertus where the reversal in elytral modifications reflects the different roles undertaken by the sexes in gallery systems with only females initiating the gallery systems and carrying out defence and maintenance activities (Kent 2001).

### Sexual differences

Fifth instar larval head capsule widths and all three adult body measurements of Austroplatypus incompertus display a size variation consistent with Bergmann’s rule which states that body size increases at higher latitudes (Blanckenhorn and Demont 2004). Latitudinal clines in body size have been observed in a number of ectotherms (Reeve et al. 2000), including arthropods (Blanckenhorn and Demont 2004). Whilst several authors (Shaw and Groeters 1998, Reeve et al. 2000) have suggested possible mechanisms for the evolution of such clines, research in this field is still in its infancy and no theory has gained ascendancy. As a result of the latitudinal cline observed in Austroplatypus incompertus, measurements for this species should be treated with caution in taxonomic works.

## Taxonomy and phylogeny

### Austroplatypus incompertus (Schedl)

Platypus incompertus, Schedl 1968, Memoirs Natural History Museum, Victoria. 28, 15.

Austroplatypus incompertus, Browne 1971, Commonwealth Forestry Review. 50, 49; Schedl, 1972a; Wood 1973, 1993; Zimmerman 1992, 1994; Kuschel 1995.

Platypus incostatus, Schedl, 1972b, Papua New Guinea Agricultural Journal. 23, 68, **syn. n.**

Platypus incompertus was described from six specimens, which Schedl thought to be male. An examination of the type material of Austroplatypus incompertus (held in the National Museum of Victoria) revealed that it consisted of females only. Platypus incompertus was subsequently placed in a new genus, Austroplatypus, by Browne (1971). His paper contains a generic description for Austroplatypus incompertus and a detailed description of its antenna, maxilla and labial palps. Browne’s miscount of the segments of the maxillary palps (four instead of three) caused problems in the taxonomic placement of platypodines within the Curculionoidea (Wood 1973, Kuschel 1995).

Armed with the knowledge that Austroplatypus incompertus displays atypical sexual dimorphism, the discrepancy between the original descriptions of both Austroplatypus incompertus (Schedl 1968) and Platypus incostatus (Schedl 1972b) may be explained. The presence of mycangia, which in platypodines are usually more developed in females than males, should have suggested to Schedl that his original six specimens were females. However, he may have decided (incorrectly) that his six specimens were males based on the structure of their elytra. Subsequent taxonomic and descriptive work (Campbell 1969, Browne 1971, Schedl 1972a, Harris et al. 1973, 1976) correctly assigned descriptions to the sexes.

Schedl (1972b) unfortunately erred again when he described Platypus incostatus as a new speciesclosely allied to Austroplatypus incompertus. Schedl distinguished this new species from Austroplatypus incompertus based mainly on the distinctly smaller size, the absence of mycangia and the reduced elytral modifications, all of which are manifestations of the atypical sexual dimorphism in this species. He was also unwilling to assign the specimen a sex.

Because the sexes in most platypodines are either similar in size or the female is slightly larger the distinctly smaller size of the single Austroplatypus incostatus specimen described by Schedl probably convinced him that he was dealing with two different species. However, the current study has shown that not only is there a significant size difference between the sexes, but also a significant size difference between beetles from different parts of their distributional range. The original type material that was used to describe Austroplatypus incompertus came from the southern part of the distribution(Eden, NSW and Woodhouse Creek north of Omeo and near Omeo Victoria), whereas the single type specimen of Platypus incostatus is from the most northern part of the distribution (Dorrigo, NSW). Thus, Schedl would have been looking at a male specimen from the smallest end of the size range, compared with female specimens from the larger end of the range. This situation would have suggested to him that he was dealing with two species.

### Position of Austroplatypus within Platypodinae

The position of Austroplatypus incompertus in the family Platypodinae is still unclear. Browne (1971) placed it in the tribe Platypodini near Dendroplatypus Browne, while Schedl (1972a) assigned it to the subfamily Platytarsilinae together with the monotypic Australian genus Notoplatypus Lea and the Malaysian genus Platytarsulus Schedl. Austroplatypus does not fit within Schedl’s Platytarsilinae, since it does not possess the distinguishing characteristics of very slender elongate body form and the proboscis-like head shape. In addition, both of the other two genera have reduced antennal segments while Austroplatypus has the four antennal segments characteristic of the rest of the platypodines (Schedl 1972a). Wood (1993) synonymised Austroplatypus under Platypus Herbst and thus also placed it in the Platypodini. He gave no reasons for this action but had possibly detected Browne’s (1971) error with respect to the number of maxillary palp segments. If so, it is strange that he did not also synonymise Dendroplatypus under Platypus, as Browne (1955) had similarly miscounted its maxillary palp as having four segments. Zimmerman (1992, 1994) and Kuschel (1995) noted Browne’s miscount of the maxillary palp segments of Austroplatypus and treated it as a valid genus, but did not comment on its classificatory position. In his review of the genus Platytarsulus Beaver (1998) also agreed that Austroplatypus was a valid genus. Since Austroplatypus incompertus differs from Platypus not only in its atypical sexual dimorphism, but alsoin its uniquely modified life-history strategies of mating and reproduction (Kent and Simpson 1992, Kirkendall et al. 1997), its placement in a separate genus within the Platypodini appears justified.

## Supplementary Material

XML Treatment for Austroplatypus incompertus

XML Treatment for Platypus incostatus
